# Isolation and characterisation of pVa-21, a giant bacteriophage with anti-biofilm potential against *Vibrio alginolyticus*

**DOI:** 10.1038/s41598-019-42681-1

**Published:** 2019-04-18

**Authors:** Sang Guen Kim, Jin Woo Jun, Sib Sankar Giri, Saekil Yun, Hyoun Joong Kim, Sang Wha Kim, Jeong Woo Kang, Se Jin Han, Dalsang Jeong, Se Chang Park

**Affiliations:** 1 Laboratory of Aquatic Biomedicine, College of Veterinary Medicine and Research Institute for Veterinary Science, Seoul National University, Seoul, Republic of Korea; 2 Department of Aquaculture, Korea National College of Agriculture and Fisheries, Kongjwipatjwi-ro, Wansan-gu, Jeonju-si, Jeollabuk-do, Republic of Korea

**Keywords:** Antimicrobials, Applied microbiology, Bacteriophages

## Abstract

There is an increasing emergence of antibiotic-resistant *Vibrio alginolyticus*, a zoonotic pathogen that causes mass mortality in aquatic animals and infects humans; therefore, there is a demand for alternatives to antibiotics for the treatment and prevention of infections caused by this pathogen. One possibility is through the exploitation of bacteriophages. In the present study, the novel bacteriophage pVa-21 was classified as *Myoviridae* and characterised as a candidate biocontrol agent against *V. alginolyticus*. Its morphology, host range and infectivity, growth characteristics, planktonic or biofilm lytic activity, stability under various conditions, and genome were investigated. Its latent period and burst size were estimated to be approximately 70 min and 58 plaque-forming units/cell, respectively. In addition, phage pVa-21 can inhibit bacterial growth in both the planktonic and biofilm states. Furthermore, phylogenetic and genome analysis revealed that the phage is closely related to the giant phiKZ-like phages and can be classified as a new member of the phiKZ-like bacteriophages that infect bacteria belonging to the family *Vibrionaceae*.

## Introduction

*Vibrio alginolyticus*, a representative of Harveyi clade bacteria, is frequently found in marine environments. This organism can infect a variety of aquatic animals and infection has been linked to several mass mortality cases in major aquaculture species from fish to molluscs and crustaceans^1–4^. Global warming has caused an increase in sea surface temperature that has undoubtedly led to the unseasonal outbreaks of *Vibrio* as well as their increased abundance and virulence in marine environments and aquaculture^5–8^. In aquatic environments, biofilms have been reported as a causative agent of disease recurrence^9,10^. Indeed, the ability of *Vibrio* spp. to form biofilms is often correlated with their pathogenicity^11,12^. Bacterial cells in biofilms are highly tolerant to antibiotics compared with those in the planktonic state^13,14^, as the biofilm matrix provides bacteria with competitive advantages for survival. Moreover, the misuse or overuse of antimicrobials has led to the emergence of multidrug-resistant bacterial strains^15^. Unsurprisingly, the isolation of *V. alginolyticus* strains with multiple antibiotic resistance have been reported from several recent outbreaks^16–18^. Therefore, there is a growing need for effective alternatives to antibiotics for managing bacterial infections and biofilms.

As viruses of bacteria, bacteriophages (phages) specifically infect and lyse targeted bacteria. Due to their specific antibacterial activities, lytic phages have been demonstrated as alternatives to antibiotic therapy in humans^19^, veterinary science^20^, and aquaculture^21^. Recently, bacteriophage VP01 showed therapeutic potential by significantly reducing the growth of *V. alginolyticus* and dispersing the biofilms in a concentration-dependent manner^22^. In addition, *V. alginolyticus* phages exhibited potential as biocontrol agents in aquatic animals such as the sea cucumber (*Apostichopus japonicus*)^23^ and brine shrimp (*Artemia salina*)^24^. In the case of biofilm-related outbreaks, phage-infected bacteria existing at the outermost region of the matrix play a pivotal role in spreading the phages through the biofilm complex. Therefore, phages have been considered as alternatives to antibiotics, especially in biofilm eradication.

In theory, bacteria in planktonic or biofilm states can be lysed by a single phage particle as its progeny can infect and lyse adjacent cells. However, phages can be affected and inactivated by environmental factors^25,26^, which may allow bacterial regrowth to occur. Therefore, it is necessary to investigate and determine the biological characteristics of phages for practical applications and to expand our understanding of phages as alternatives to antibiotics.

With the goal of improving treatment and prevention of *V. alginolyticus* infection, we isolated and characterised a new lytic phage infecting *Vibrio* strains. This study focuses on the anti-planktonic and anti-biofilm activities of this phage and its genomic properties.

## Results

### Isolation and biological properties of phage pVa-21

**Figure 1:**
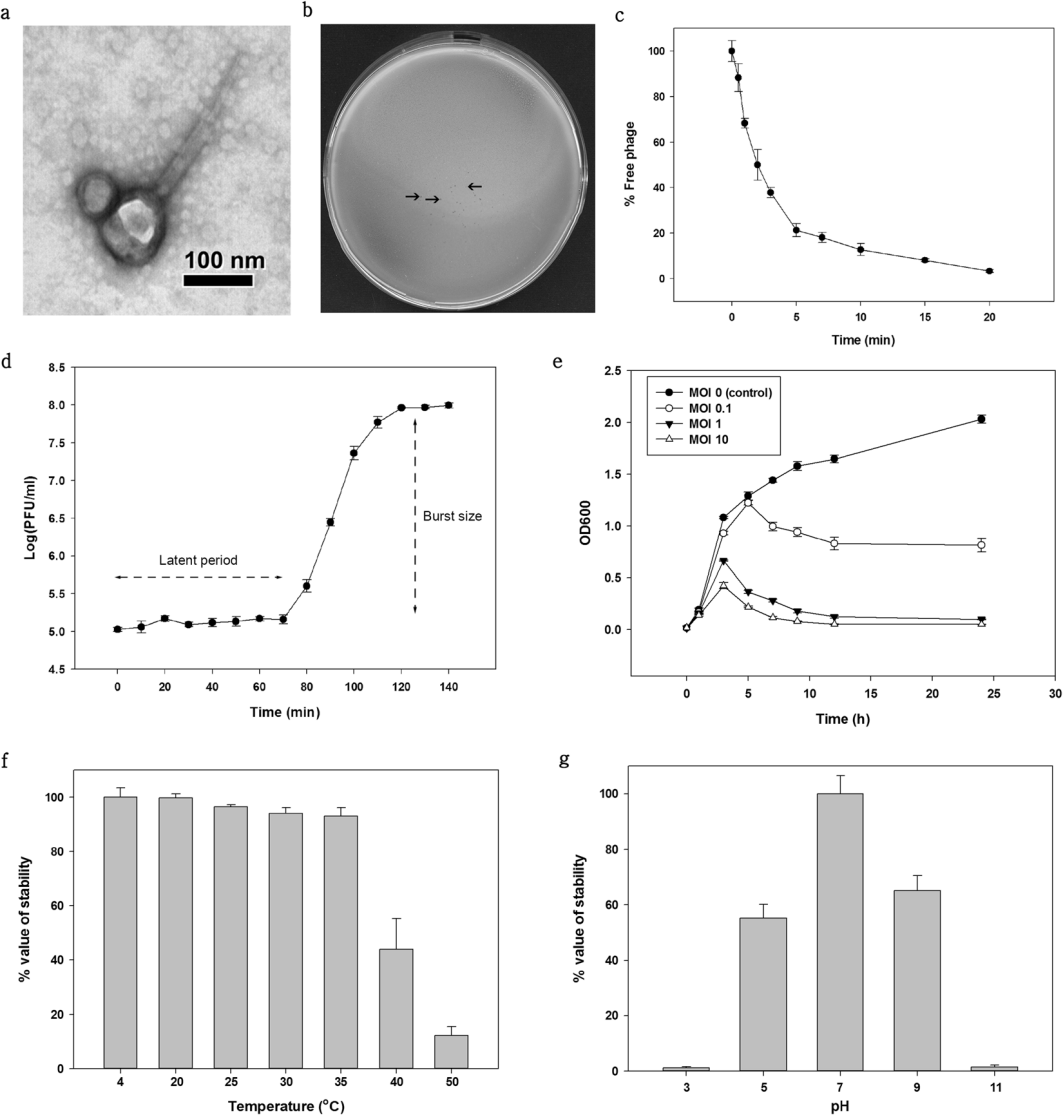
Morphology and biological properties of phage pVa-21. (**a**) Transmission electron micrograph of pVa-21. Scale bar = 100 nm. (**b**) Plaque morphology of phage pVa-21. The arrow represent plaques. (**c**) Adsorption assays of pVa-21 with *V. alginolyticus* strain rm-8402. (**d**) One-step growth curve of pVa-21 in culture broth of *V. alginolyticus* strain rm-8402. (**e**) Planktonic cell lysis kinetics of pVa-21 at an MOI of 0.1, 1, and 10 on *V. alginolyticus* strain rm-8402. (**f**) Thermal stability of pVa-21. Phages were incubated for 1 h under different temperatures. (**g**) pH stability of pVa-21. Phages were incubated for 1 h under different pH values. (**f**,**g**) Phage ability to form plaques on host lawns was determined. Relative to control, changes in PFUs were calculated. The results shown in (**c–g**) represent the mean ± standard deviation of triplicate experiments.

*V. alginolyticus* phages were isolated from seawater samples after enrichment. Purified phages were examined by transmission electron microscopy (TEM) and classified based on the criteria proposed by Ackermann^27^. As shown in Fig. 1a, phages were designated as pVa-21 and assigned to the family *Myoviridae*. It possesses an icosahedral head 87 ± 3 nm in diameter (n = 5) and a contractile tail 240 ± 9 nm in length (n = 5). The host range test was determined against bacteria of the Harveyi clade, which includes major pathogens of aquatic organisms, including *V. alginolyticus* (n = 5), *V. harveyi* (n = 5), *V. parahaemolyticus* (n = 1), *V. anguillarum* (n = 1), *V. campbellii* (n = 1), and *V. vulnificus* (n = 1). Phage pVa-21 was able to infect *V. alginolyticus* (n = 3) and *V. harveyi* (n = 1; Table 1). However, the phage did not show infectivity against the ten other bacterial strains tested and phage pVa-21 plaques were very small in size (less than 1 mm; Fig. 1b). The efficiency of plating (EOP) value varied among the *Vibrio* species, and no strain showed a higher value than the indicator host strain, *V. alginolyticus* rm-8402. Therefore, *in vitro* phage infection kinetics on host *V. alginolyticus* rm-8402 were assessed for adsorption rate, one-step growth curves, and cell lysis at two different multiplicities of infection (MOIs). The percentage of adsorption on the tested strain was 95% after 15 min (Fig. 1c). To identify the growth pattern and burst size of pVa-21, a one-step growth curve was generated. The latent period was found to be approximately 70 min and the burst size, i.e., the number of progeny released after lysis of a single bacterial cell, was approximately 58 virions per cell (Fig. 1d). Kinetics of the planktonic cell lysis test was conducted as shown in Fig. 1e. Strains that were not inoculated with phage pVa-21 (MOI  = 0) showed a continuous increase in optical density (OD)_600_ values during the 24 h of incubation. When the lowest concentration (MOI  = 0.1) of phage was applied, the bacterial strain showed partial lysis and growth reached OD_600_ = 1.0 (7.5 × 10^8^ colony forming units [CFU]/mL) at 24 h; meanwhile, the control group showed OD_600_ = 2.0 and 1.4 × 10^9^ CFU/mL. In contrast, bacterial growth increased until 3 h and was then completely lysed by the phage at an MOI of 1 and 10. Furthermore, pVa-21 stability was tested at different pH values and temperatures and was estimated by determining the changes in growth based on the number of plaque-forming units (PFU). Short-term thermal stability tests showed that pVa-21 was stable at 4 °C (control), 20 °C, 25 °C, 30 °C, and 35 °C for 1 h, but phage numbers were significantly lower at 40 °C (p < 0.001) and 50 °C (p < 0.001; Fig. 1f). Additionally, pVa-21 showed a significant decrease (p < 0.001) in PFUs under all pH treatment groups except at pH 7 (control group; Fig. 1g).

**Table 1:** Host range of phage pVa-21 against all bacterial strains used in this study.

Bacterial species	Strain	EOP^a^	Source
*V. alginolyticus*	rm-8402	1.00	Takaoka *et al*.^2^
	V374	0	Nishibuchi *et al*.^58^
	V447	0.76 ± 0.09	Sawabe *et al*.^59^
	am-10	0.15 ± 0.12	Seoul National University Aquatic Biomedicine Laboratory Culture collection
	SNUFPC 080402	0	″
*V. harveyi*	SFC-BS	0.70 ± 0.03	″
	PG-9302	0	″
	PG-9303	0	″
	O-3	0	Ishimaru and Muroga^60^
	O-6	0	Ishimaru and Muroga^60^
*V. parahaemolyticus*	CRS 09–17	0	Jun *et al*.^61^
*V. anguillarum*	HT7601	0	Nishibuchi *et al*.^58^
*V. campbellii*	HUFP 9109	0	Hiroshima University Fish Pathology Laboratory Culture Collection
*V. vulnificus*	HM1-1	0	Nishibuchi *et al*.^58^

^a^EOP value is represented as the means ± standard deviation of triplicate replicates.

EOP; efficiency of plating.

### Biofilm treatment with phage pVa-21

**Figure 2:**
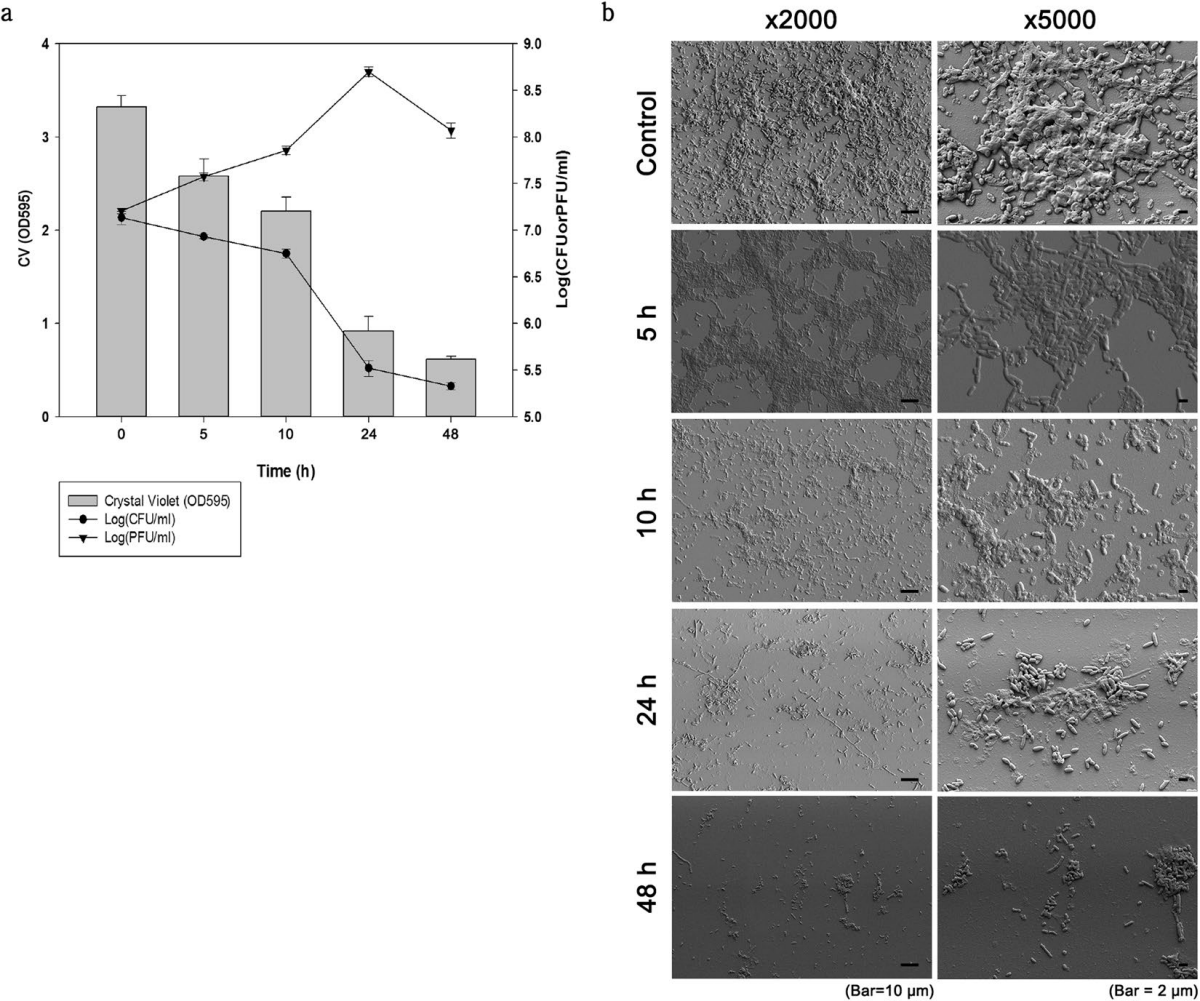
Infection dynamics of phage pVa-21 on biofilms of *V. alginolyticus* strain rm-8402. (**a**) Biofilms (48 h old) formed in 96-well plates were treated with pVa-21 at concentrations of 10^7^ PFU/mL. Total biofilm biomass was measured at OD_595_ and stained with 1% crystal violet. Viable bacterial cell and phage counts were measured by direct plating on agar. The results represent the mean ± standard deviation of triplicate experiments. (**b**) Scanning electron micrograph of strain rm-8402 biofilm formation on glass coverslips. Scale bar = 10 μm and 2 μm at 2000× and 5000× magnification, respectively.

*V. alginolyticus* strain rm-8402 was left to form a biofilm for 48 h and then treated with phage pVa-21 (1.6 × 10^8^ PFU/mL). Changes in CFUs, PFUs, and total biofilm biomass by crystal violet staining were measured over a 48-h period following infection. Total biofilm biomass showed a significant reduction (p < 0.001) after 5 h of phage treatment and the viable bacterial cell count inside the biofilm was significantly decreased (p < 0.001) after treatment for 5 h or more (Fig. 2a). During the first 24 h, crystal violet staining intensity and viable cell counts declined rapidly and did not increase over the next 24 h, indicating no biofilm regrowth. As bacterial cell counts decreased after phage treatment, phage concentration increased by more than 10-fold after 24 h compared with its initial concentration. After 48 h, phage concentrations decreased slightly but maintained a concentration of 1.19 × 10^8^ PFU/mL (Fig. 2a). Scanning electron microscopy (SEM) verified that *V. alginolyticus* rm-8402 formed a biofilm (Fig. 2b) and revealed that the biofilm could only exist within 10 h after phage treatment. At first, bacterial cells located at the edge of the biofilm were disrupted by the phage and washed away after 5 h. Next, the film structure began to disappear at 10 h and then small bacterial aggregates, rather than film structure, were observed at 24 and 48 h after phage treatment (Fig. 2b).

### Genomic characterisation of phage pVa-21

**Figure 3:**
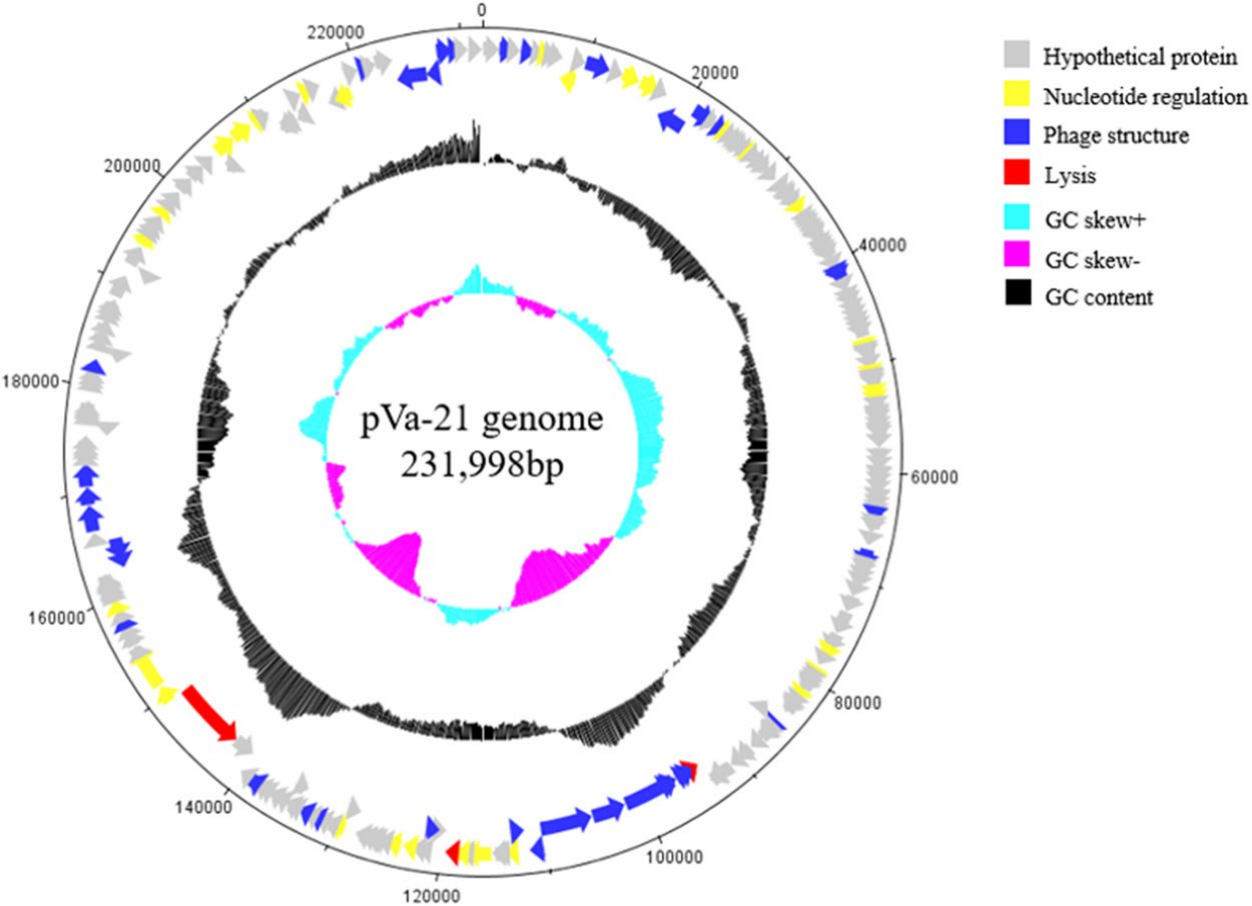
Genome map of phage pVa-21. The innermost circles coloured in cyan and purple indicate the positive and negative GC skew, respectively. Black circle indicates GC content. The functional categories of ORFs are indicated by specific colours; grey ORFs represent hypothetical proteins, yellow ORFs represent nucleotide regulation proteins, blue ORFs represent structure and packaging proteins, and red ORFs represent lysis proteins. Scale units are base pairs.

The whole genome of phage pVa-21 was sequenced and analysed. Generally, genomes of phages belonging to the family *Myoviridae* consist of double-stranded DNA (dsDNA)^28^. In line with this, pVa-21 genomic DNA was digested by DNase I but not RNase A, indicating that it is a DNA phage (data not shown). The complete genome sequence of pVa-21 was 231,998 bp long with a GC content of 44.58%, encoding 241 putative open reading frames (ORFs) and no tRNA genes. As shown in Fig. 3, the ORFs of pVa-21 were broadly scattered across the genome and were not clustered according to function, such as those encoding structural, metabolism-related, or lysis proteins. Functional examination of the predicted ORFs indicated that they could be classified into three main categories, nucleotide regulation (e.g., helicase, ribonuclease H, DNA-directed RNA polymerase beta subunit), structure and packaging (e.g., major capsid protein, terminase large subunit, tail fibre protein), and lysis (e.g. lytic transglycosylase). Most genes (211; 87.5%) were located on the positive strand with only 30 genes (12.5%) located on the negative strand. Table S1 lists the general features of the putative ORFs identified in pVa-21.

**Figure 4:**
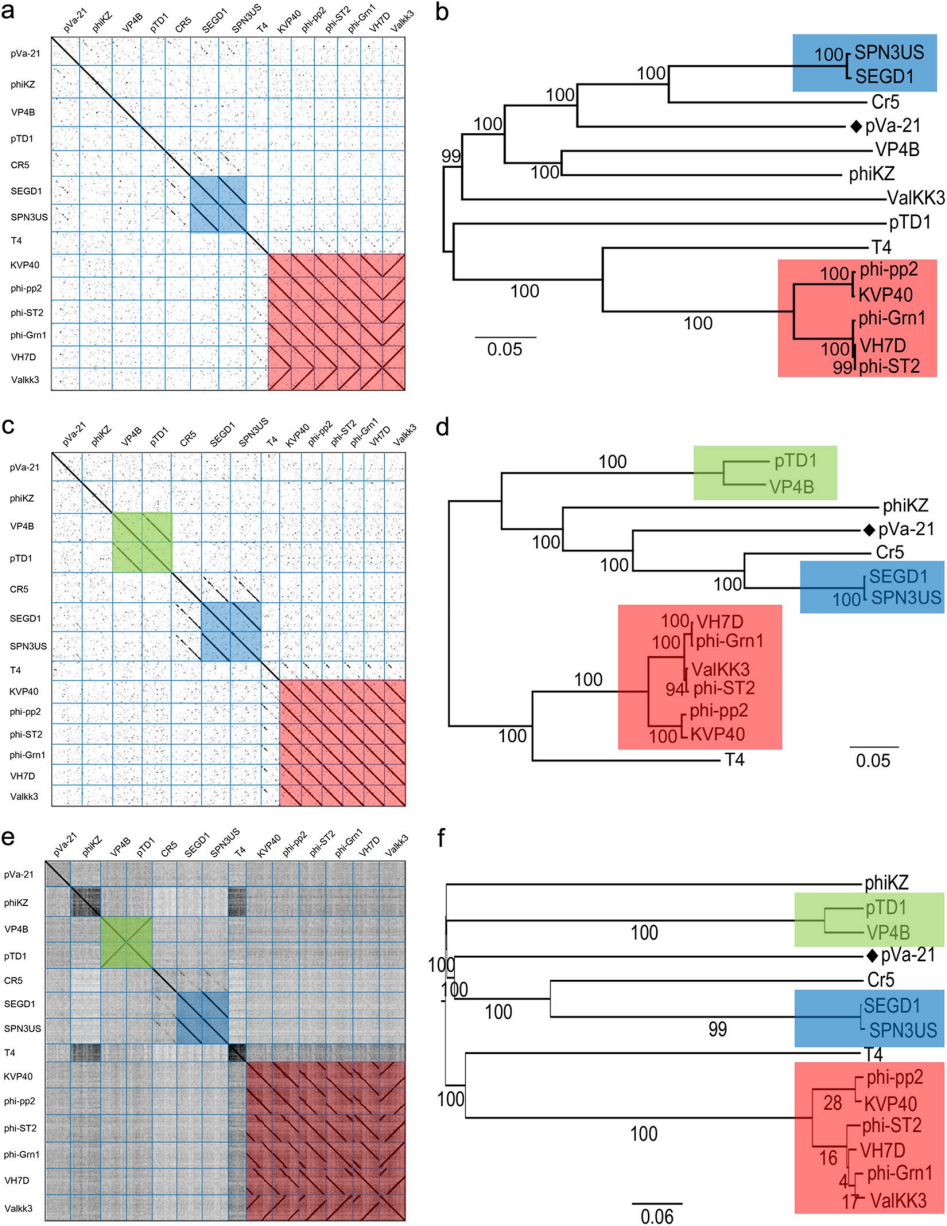
Comparative analysis of the phage pVa-21. Analysis was conducted using (**a**,**b**) terminase large subunit sequences, (**c**,**d**) major capsid protein sequences, (**e**,**f**) and whole genome sequences. The dot plot was generated in Gepard^52^ at a word size of 10. The phylogenetic tree was generated in MEGA 7.0 software^54^ using (**b**,**d**) the neighbour-joining method and (**e**) VICTOR^57^ with settings recommended for prokaryotic viruses. (**a–e**) Clustered phages are indicated with different coloured boxes. Blue box, phiKZ-like *Enterobacteria* phage SEGD1 and SPN3US; green box, phiKZ-like *Vibrio* phage VP4B and pTD1; red box, schizo T4-like phages.

A BLASTn search revealed that phage pVa-21 is related to the phiKZ-like phage group (>65% similarity), which includes the *Salmonella* phage SPN3US, *Cronobacter* phage CR5, and enterobacteria phage SEGD1. Comparative analysis was then conducted using phylogeny and the dot plot method (Fig. 4). Terminase large subunit, major capsid protein, and whole genome sequences were used to determine phage relatedness. Dot plot results showed a strong relationship among schizo T4-like phages, while phiKZ-like phages showed clustering; cluster 1 included VP4B and pTD1 and cluster 2 included SPN3US and SEGD1 (Fig. 4a,c,e). Phage pVa-21, newly isolated in this study, showed a weak diagonal pattern with SPN3US, CR5, and SEGD1 for both major capsid protein and terminase large subunit sequences; however, no patterns with T4 and schizo T4-like phages were observed (Fig. 4a,c). The whole genome plot showed clustering as shown on the plot using major capsid protein and terminase large subunit sequences; however, no patterns were observed between pVa-21 and other related phages (Fig. 4e). Phylogenetic analysis revealed that pVa-21 was closely related to the phiKZ-like phages SPN3US, CR5, and SEGD1 (Fig. 4b,d,f) and distantly related to the *Pseudomonas* bacteriophage phiKZ. As revealed through dot plot analysis, three clusters were generated (SPN3US and SEGD1; pTD1 and VP4B; and schizo T4-like phages). Interestingly, the schizo T4-like *Vibrio* phage ValKK3 was found clustered with phiKZ-like phages after analysis using terminase large subunit sequences (Fig. 4b).

By using the artemis comparison tool (ACT), we were able to visualise whole genome comparisons of pVa-21 with VP4B (phiKZ-like *Vibrio* phage) and KVP40 (schizo T4-like *Vibrio* phage) as representatives of each group (Fig. S1); homology intensity was found slightly higher with VP4B. Nucleotide homology was also analysed using terminase large subunit, major capsid protein, and whole genome sequences. As shown in Table S2, nucleotide homology was higher with phiKZ-like phages than with schizo T4-like phages based on the terminase large subunit and major capsid protein sequences. Moreover, the gap value between pVa-21 and other phages was shorter with the phiKZ-like phages. Whole genome-wide comparisons, however, revealed no differences in percent identity and gap value. Furthermore, we compared the proteome of phage pVa-21 with two PhiKZ-like *Vibrio* phages and six schizo T4-like *Vibrio* phages using CoreGene. A total of 61 proteins were shared with phiKZ-like *Vibrio* phages while 12 proteins were shared with schizo T4-like *Vibrio* phages (Table S3).

Overall, the results of these comparative genomic analyses indicate that phage pVa-21 is more homologous with the phiKZ-like phage group rather than the schizo T4-like phage group and can be considered a distant member of phiKZ-like phages. Moreover, several paralogous genes similar to the beta/beta′ subunit of RNA polymerase were found in the pVa-21 genome, which is a distinctive feature of phiKZ-like phages^29,30^. In addition, three lysis-related proteins were identified in the pVa-21 genome, locus tag pVa21_119 (AQT28060.1), pVa21_134 (AQT28075.1), and pVa21_165 (AQT28106.1). Locus tag pVa21_119, pVa21_134, and pVa21_165 possessed lysis catalytic domains similar to the glycosyl hydrolase 108 domain, lytic transglycosylase domain, and goose egg white lysozyme domain, respectively. All lysis-related proteins were well conserved in phiKZ-like phages while pVa21_165 (AQT28106.1) was found in all phiKZ- and schizoT4-like *vibrio* phages analysed in this study. Additionally, genomic DNA of phage pVa-21 was examined for the presence of antimicrobial resistance genes, but no such gene was identified.

## Discussion

In aquatic environments and fisheries, unseasonal outbreaks of *Vibrio* spp. are increasing due to global warming. In some cases, bacteria in biofilms showed increased resistance against antibiotics, which is a major obstacle to the current treatment method as it relies on antibiotics. Several studies have also reported a relationship between biofilms and disease recurrence. Notably, *V. alginolyticus* can form biofilms on polyvinyl-chloride, polyethylene, polystyrene, and glass surfaces that are generally used in aquaculture installations. Consequently, the efficacy of antibiotic therapy is decreasing and new methods are needed in place of antibiotics to control biofilms and multidrug-resistant bacteria effectively.

There have been several reports on controlling *Vibrio* spp. using bacteriophages^21–24,31^. However, limited reports are available regarding phages that can infect *V. alginolyticus* and control biofilm formation, even though this bacterial species can infect a variety of aquatic animals and even humans. In this study, we isolated the novel phage pVa-21, which infects *V. alginolyticus*, and characterised its biological properties. Phage pVa-21 can infect several fish pathogenic Harveyi clade bacteria. Moreover, pVa-21 was found to be stable up to 35 °C and at a pH range of 7–9, making it suitable for use under normal seawater conditions (i.e., 0–35 °C and pH 7.5–8.4).

As a biocontrol agent, phage therapy should also consider the emergence of resistance as observed for antibiotics. One of the most critical aspects is controlling microbial regrowth. Phage pVa-21 showed considerable anti-planktonic and anti-biofilm effects where planktonic bacteria were lysed after treatment with pVa-21 at an MOI of 1 or 10 (Fig. 1e). Moreover, biofilms were considerably disrupted and did not show regrowth; instead, viable bacterial cells remained in small aggregates (Fig. 2). At an MOI = 0.1, bacterial regrowth was observed. Similarly, a previous study also observed bacterial regrowth after phage treatment, yet additional inoculation with the phage solution could control these regrown bacteria as not all bacteria were resistant to the phages^32^. Another study suggested that regrown bacterial cells recover susceptibility when subsequently cultured in a phage-free medium, illustrating the transiency of phage resistance^33^. Indeed, phage pVa-21 formed inhibition zones on lawns of regrown bacteria when spot assays were conducted (data not shown), confirming that susceptiblity of the regrown bacteria and effectiveness of the giant vibriophage pVa-21 were maintained. However, follow up studies should address how pVa-21 prevents *V. alginolyticus* regrowth and controls mature biofilms for future application in environmental or industrial settings.

The anti-biofilm effect of phages and their enzymes has been previously demonstrated^34^. A recent study demonstrating the anti-biofilm effect of a phiKZ-like phage infecting *V. alginolyticus* supports the possibility of implementing phage pVa-21 as a biocontrol agent^35^. However, owing to specificity, phages cannot control a pathogen if it is not susceptible to the phage. To offset this disadvantage, isolating various types of phages with different host ranges and implementing cocktail therapy to expand antibiotic activity against target pathogens is imperative.

In our study, genetic characterisation revealed that pVa-21 was similar to phiKZ-like phages. Several beta/beta′ RNA polymerase subunits are characteristic of phiKZ-like phages^29,30^, and phage pVa-21 possessed several virion-associated beta/beta′ RNA polymerase subunits homologous to those of phiKZ. Phylogenetic analysis led to phage pVa-21 clustering with phiKZ-like phages rather than with schizo T4-like phages (Fig. 4). Dot plot and nucleotide homology results between pVa-21 and phiKZ-like *Vibrio* bacteriophages or schizo T4-like *Vibrio* bacteriophages also supported that pVa-21 is closely related to the phiKZ-like group rather than the schizo T4-like phage group. Furthermore, pVa-21 was found to exhibit small plaque sizes, which is also a characteristic of phiKZ-like phages^36^. Based on the currently available databases, no antibiotic resistance, virulence, or temperate phage-related genes were detected in the genome, suggesting that the *V. alginolyticus* phage pVa-21 can be safely used as a biocontrol agent. However, the majority of ORFs in pVa-21 did not match with predicted functions in GenBank. Thus, to ensure the safe and reliable application of phages in therapeutic settings, further investigation of the phage genome is warranted to gain a deeper understanding of the roles of encoded gene products, as they may produce novel virulence factors or interact undesirably with the host genome^37^.

In conclusion, the present study characterised the novel phiKZ-like phage pVa-21. Planktonic bacterial cell lysis and biofilm disruption effects of this *Vibrio* phage help promote the application of bacteriophages in biofilm control. Moreover, the pVa-21 genome is expected to broaden the phiKZ-like phage library. Although the precise biofilm eradication mechanism of pVa-21 remains to be elucidated, our findings support the potential of pVa-21 in controlling the fish pathogen *V. alginolyticus*. Further studies are needed to isolate other phages for effective pathogen control.

## Materials and Methods

### Bacterial strains and growth conditions

All bacterial strains used in this study are listed in Table 1. Bacteria were cultured in tryptic soy broth (TSB; Becton Dickinson, Franklin Lakes, NJ) supplemented with 1.5% (w/v) sodium chloride (Daejung Chemicals, Gheonggi-do, South Korea) with shaking at 150 rpm or sub-cultured on tryptic soy agar (Becton Dickinson) at 27 °C.

### Phage isolation, purification, and propagation

To isolate phages infecting *V. alginolyticus*, 65 water samples from the West Sea of South Korea were collected over the span of five months and filtered through 0.2-µm membrane filters (Merck Millipore, Burlington, MA). *V. alginolyticus* strain rm-8402, which was previously reported as a fish pathogen^2^, was used as an indicator host strain. To isolate phages, 1% (v/v) of overnight grown (early stationary phase) host strain rm-8402 was used to inoculate a mixture of collected seawater samples and TSB (1:1) and cultured for 24 h at 27 °C. After enrichment, the presence of the phage was verified by spotting the serially diluted culture broth on the bottom agar layered with bacteria. Culture samples that showed inhibition zones were centrifuged at 10,000 × *g* for 20 min and the resulting supernatant was filtered through a 0.2-μm membrane filter. To confirm the presence of the lytic phage in the filtrate, a double-layer agar method was performed using the filtrate^23^. After overnight incubation at 27 °C, the plaque was purified five times through single-plaque isolation with a sterile straw to ensure that the isolated phages were descendants from a single virion. The *Vibrio* phages formed clear and small plaques on *V. alginolyticus* rm-8402 lawns (Fig. 1a) and were thus selected for further study and designated pVa-21.

### Electron microscopy

For phage TEM, the obtained phages were concentrated using polyethylene glycol 8000-NaCl precipitation in sodium chloride-magnesium sulfate (SM) buffer (100 mM NaCl, 50 mM Tris pH 7.5, and 10 mM MgSO_4_) and then 10 μL of the suspension was spotted on a copper grid. After 2 min, the suspension was removed by absorption onto filter paper and the phages were negatively stained with 2% uranyl acetate for 1 min, followed by three successive washes with water. The grid was air-dried for 10 min and then imaged with a JEM-1010 (Jeol, Tokyo, Japan) operated at 80 kV. Phage dimensions were calculated by measuring the dimensions of five independent phages. The biofilm degradation effects of phage pVa-21 were observed using a ZEISS Sigma field-emission scanning electron microscope (FE-SEM; Carl Zeiss, Oberkochen, Germany) operated at 15 kV. Each biofilm was washed with phosphate-buffered saline (PBS), fixed with 2.5% glutaraldehyde for 1 h, and dehydrated in a graded series of ethanol (50%, 70%, 90%, and 95%; 1 h per step) and three times in 99% ethanol for 1 h. The biofilm was then dried with vacuum desiccator overnight and coated with platinum.

### Host range analysis

The host range of the obtained phage was determined using a spot assay and confirmed by the double-layer agar method. Ten microliters of the phage lysate (>10^7^ PFU/mL) was dropped onto the overlaid top agar and mixed with each bacterial strain. The plates were then incubated overnight at 27 °C and checked for the presence of a lysis zone. An EOP assay was conducted to quantify the lytic activity of phage pVa-21. The phage suspension (10^3^ PFU/mL) was then assayed by the double-layer agar method. The total number of plaques was determined after 24 h of incubation and EOP values were calculated by comparing the ratios of PFUs of a susceptible strain to the indicator strain rm-8402 in triplicate.

### Adsorption assay and one-step growth curve

The adsorption assay was carried out as described by Lu *et al*.^38^. The exponentially growing host strain (1.5 × 10^8^ CFU/mL) was infected with a phage suspension at an MOI (the ratio of virus to bacterial cells) of 0.001 and incubated at 27 °C. Aliquots (100 μL) were taken at 0, 0.5, 1, 2, 3, 5, 7, 10, 15, and 20 min after infection and immediately diluted in 900 μL PBS, followed by centrifugation at 12,000 × *g* for 5 min. The supernatants were titrated for un-adsorbed free phages using the double-layer agar method. To construct a growth curve, the phage lysate was used to inoculate 10 mL of exponentially growing host strain culture (1.5 × 10^8^ CFU/mL) at an MOI of 0.001. The phage was absorbed for 15 min and then centrifuged at 12,000 × *g* for 5 min. After the supernatant was discarded, the phage-infected bacterial pellet was re-suspended in 10 mL of preheated TSB and incubated at 27 °C with shaking at 250 rpm. At 10 min intervals, 100-μL aliquots were taken until 140 min and then titres were immediately determined by the double-layer agar method. Titre measurements were carried out in triplicate.

### pH and thermal stability assays

For pH stability tests, 10 μL phage suspension (1.3 × 10^9^ PFU/mL) was used to inoculate 1 mL PBS adjusted to pH 3.0, 5.0, 7.0, 9.0, and 11.0 with 1 M NaOH or 1 M HCl. The tubes were then incubated at 27 °C and aliquots were taken after 60 min. For thermal stability tests, 1 mL phage suspension (1.3 × 10^7^ PFU/mL) was incubated at 4 °C, 20 °C, 25 °C, 30 °C, 35 °C, 40 °C, and 50 °C and then 100-μL aliquots were collected after 60 min. Next, aliquot titres were calculated using a 10-fold serial dilution. All tests were performed in triplicate.

### Bacterial cell lysis assay

To evaluate the bacteriolytic efficacy of phage pVa-21, bacterial strains showing a turbid or clear lysis pattern in the spot assay were selected. One percent of overnight culture was inoculated into 10 mL of fresh broth to obtain 10^8^ CFU/mL and then the phage was used to inoculate the broth at an MOI of 0, 0.1, 1, and 10. The broth was cultured with vigorous shaking and then OD_600_ was measured at 0, 1, 3, 5, 7, 9, 12, and 24 h. All tests were performed in triplicate.

### Biofilm treatment with pVa-21

To verify the anti-biofilm efficacy of pVa-21, a biofilm was formed according to a previously described method with minor modifications^39^. Briefly, the biofilm assay was performed in 96-well polystyrene tissue culture microplates (Nunc, Roskilde, Denmark). One percent of overnight culture was inoculated into fresh TSB supplemented with 1% d-glucose (Sigma-Aldrich, St. Louis, MO) and then aliquots (200 μL) were distributed to each microplate well. Two sets of microplates—one for staining and the other for enumeration of bacteria or bacteriophages—were then incubated at 27 °C for 48 h with no shaking. The supernatant of each well was removed and washed twice with PBS to remove all planktonic cells, followed by treatment with 200 μL phage suspension (1.6 × 10^8^ PFU/mL) for 3, 5, 7, 10, 24 and 48 h; changes in PFU were enumerated using the supernatant of each time point. The microplates were then washed twice with PBS and dried. To enumerate viable bacterial cells, biofilm cells were re-suspended in PBS via scraping with a sterile tip and then the suspension was diluted and plated. To quantify the total biomass, formed biofilms were stained with 1% crystal violet for 15 min, after which the wells were washed once more. Crystal violet was dissolved in an ethanol-acetone solution (80:20 v/v) and OD was measured at 595 nm. For SEM, biofilms were formed on glass coverslips (22 × 22 mm) submerged in media in 6-well plates and incubated for 48 h at 27 °C. Then, planktonic cells were removed from the wells, washed with PBS, and inoculated with phage suspension (10^8^ PFU/mL) for 3, 5, 7, 10, 24, and 48 h. At each time point, the glass coverslips were processed as mentioned above and then SEM was performed.

### Phage sequencing and genome analysis

Phage genomic DNA was extracted as described previously with minor modifications^40^. Briefly, the bacteriophage lysate was treated with 10 U DNase I and RNase A (Takara Bio, Kyoto, Japan) to degrade genomic DNA and RNA of the *V. alginolyticus* host cells according to manufacturer’s instructions. Then, ethylenediaminetetraacetic acid (EDTA) was added to inhibit nucleases. Protease K was also added and incubated at 37 °C for 30 min and then inactivated at 95 °C for 15 min. DNA purification followed conventional phenol-chloroform extraction methods^41^.

Purified genomic DNA of the phage was then sequenced using an Illumina HiSeq2500 platform (Illumina, San Diego, CA) at Genotech (Daejeon, South Korea). Reads were trimmed and assembled using the CLC Genomic Workbench v6.5.1. Putative ORFs were predicted and annotated using Glimmer v3.02^42^, Prodigal v1.20^43^, and protein BLAST. The Rapid Annotation using Subsystem Technology (RAST) server was used for confirmation^44^. Detection of tRNAs was carried out using tRNAscan-SE v2.0^45^ and the genome map of pVa-21 was drawn using DNA plotter^46^. The web tool RESFINDER v2.1 was used to search for known antimicrobial resistance coding genes^47^. Percent nucleotide homology was calculated using EMBOSS Stretcher^48^ and protein sequence similarities of the phages were analysed using CoreGenes3.5 software^49^ with the default setting. A dot plot was generated in Gepard^50^ at a word size of 10.

For phylogenetic analysis, amino acid sequences of the major capsid protein and terminase large subunit were obtained from the Genbank database and aligned using Clustal W^51^. A phylogenetic tree was constructed using the neighbour-joining method implemented in MEGA v7.0^52^ with 1000 bootstrap replications. The whole genome phylogenetic tree was generated in the Virus Classification and Tree Building Online Resource (VICTOR)^53^ using the Genome-BLAST Distance Phylogeny (GBDP) method^54^ under settings recommended for prokaryotic viruses^53^. The resulting intergenomic distances (including 100 replicates each) were used to infer a balanced minimum evolution tree with branch support via FASTME including Subtree Pruning and Regrafting (SPR) postprocessing^55^ for the formula D0. The tree was rooted at the midpoint^55^ and visualised with FigTree^56^.

### Statistical analysis

All analyses were performed with SigmaPlot 12.0 software (Systat Software, Inc. Chicago, IL) using ANOVA with Dunnett’s post-hoc test. P values < 0.05 were considered statistically significant.

### Nucleotide sequence accession numbers

The genome sequence of the isolated phage pVa-21 was deposited in GenBank under the accession number KY499642.

## Supplementary information

Supplementary Information

## Data Availability

All data generated or analysed during this study are included in this published article and its supplementary information files.
