# Supplementary material for: Isolation and characterisation of pVa-21, a giant bacteriophage with anti-biofilm potential against Vibrio alginolyticus
Source: Sci Rep. 2019 Apr 18;9:6284. doi: 10.1038/s41598-019-42681-1 (PMC6472347; doi:10.1038/s41598-019-42681-1)
Supplement: Supplementary file 1 — Supplementary Information [file 41598_2019_42681_MOESM1_ESM.docx]

**Supplementary Information**

**Isolation and characterization of pVa-21, a giant bacteriophage with anti-biofilm potential against *Vibrio alginolyticus***

Sang Guen Kim^1^, Jin Woo Jun^2^, Sib Sankar Giri^1^, Saekil Yun^1^, Hyoun Joong Kim^1^, Sang Wha Kim^1^, Jeong Woo Kang^1^, Se Jin Han^1^, Dalsang Jeong^2^, Se Chang Park^1*^

^1^ Laboratory of Aquatic Biomedicine, College of Veterinary Medicine and Research Institute for Veterinary Science, Seoul National University, Seoul, Republic of Korea

^2^ Department of Aquaculture, Korea National College of Agriculture and Fisheries, Kongjwipatjwi-ro, Wansan-gu, jeonju-si, jeollabuk-do, Republic of Korea

* E-mail: parksec@snu.ac.kr (SCP)

**Figure S1** Comparative genome analysis of phage pVa-21**.** Whole genomes of KVP40 (NC_005083), schizo T4-like phages, pVa-21 (KY499642), and VP4B (KC131130), phiKZ-like phages was compared using artemis comparison tool (ACT).


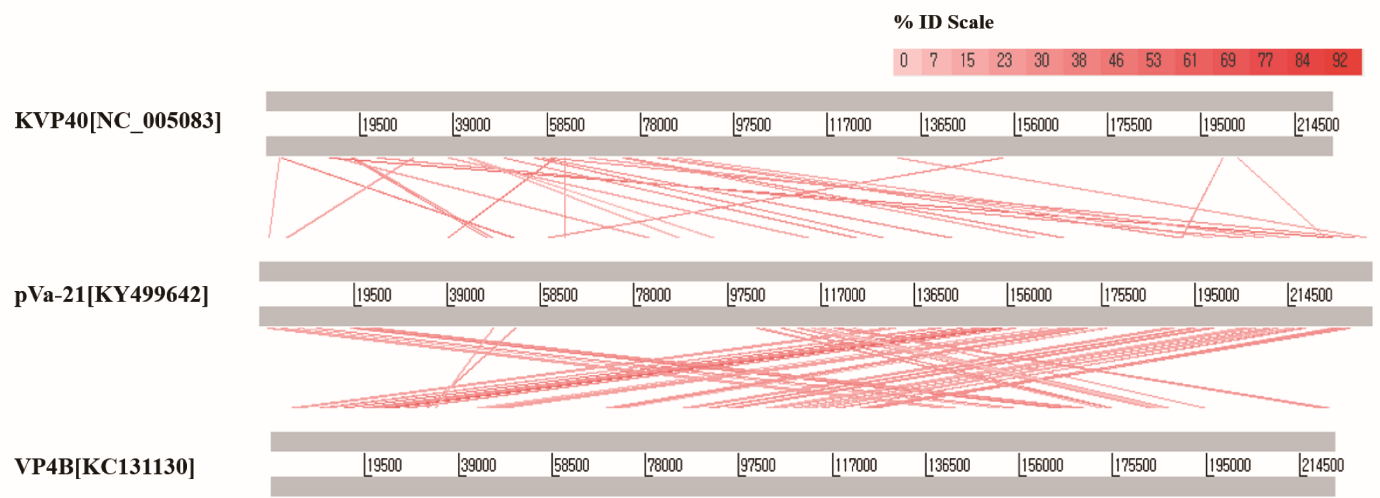


**Table S1** Functional categories of the predicted open reading frames (ORFs) in bacteriophage pVa-21.

| **Group** | **Subgroup** | **Locus tag** | **Encoded protein** | **Related organism** | **Identity (%)** |
| --- | --- | --- | --- | --- | --- |
| Structure and packaging |  | pVa21_002 | Putative virion structural protein | *Cronobacter* phage CR5 | 30 |
| Structure and packaging |  | pVa21_004 | Putative virion structural protein | *Erwinia* phage vB_EamM_Asesino | 29 |
| Nucleotide regulation |  | pVa21_006 | Transcriptional regulator | *Rhodococcus* *opacus* | 38 |
| Nucleotide regulation | replication | pVa21_009 | Putative helicase | *Cronobacter* phage CR5 | 39 |
| Structure and packaging | head | pVa21_011 | Putative major capsid protein | *Cronobacter* phage CR5 | 37 |
| Nucleotide regulation |  | pVa21_013 | Putative RNA polymerase beta subunit | *Erwinia* phage vB_EamM_Kwan | 27 |
| Structure and packaging |  | pVa21_016 | Putative virion structural protein | *Erwinia* phage vB_EamM_EarlPhillipIV | 33 |
| Structure and packaging |  | pVa21_017 | Putative virion structural protein | *Erwinia* phage phiEaH2 | 34 |
| Structure and packaging |  | pVa21_019 | Putative virion structural protein | *Cronobacter* phage CR5 | 42 |
| Nucleotide regulation | replication | pVa21_020 | Putative crossover junction | *Erwinia* phage vB_EamM_Huxley | 35 |
| Nucleotide regulation |  | pVa21_026 | N-acetyltransferase | *Enterococcus rotai* | 36 |
| Nucleotide regulation |  | pVa21_035 | DNA recombination-mediator protein A | *Pseudomonas* phage VCM | 32 |
| Structure and packaging |  | pVa21_047 | putative virion structural protein | *Cronobacter* phage CR5 | 28 |
| Structure and packaging |  | pVa21_048 | putative virion structural protein | *Cronobacter* phage CR5 | 42 |
| Nucleotide regulation |  | pVa21_057 | Thymidylate synthase | *Erwinia* phage vB_EamM_Kwan | 31 |
| Nucleotide regulation |  | pVa21_061 | GTP pyrophosphokinase | *Burkholderia multivorans* | 46 |
| Nucleotide regulation |  | pVa21_065 | ADP-ribose pyrophosphatase | *Cellvibrio* sp. BR | 44 |
| Nucleotide regulation |  | pVa21_066 | dihydrofolate reductase | Bacteroidetes bacterium GWE2_32_14 | 39 |
| Structure and packaging |  | pVa21_085 | prohead core scaffolding protein | *Enterobacteria* phage vB_KleM-RaK2 | 33 |
| Structure and packaging | tail | pVa21_089 | putative tail fiber protein | *Erwinia* phage vB_EamM_EarlPhillipIV | 41 |
| Nucleotide regulation |  | pVa21_098 | transcriptional regulator | *Vibrio* sp. ER1A | 56 |
| Nucleotide regulation |  | pVa21_100 | thymidylate kinase | *Xenorhabdus bovienii* | 40 |
| Nucleotide regulation |  | pVa21_105 | deoxyuridine 5’-triphosphate nucleotidohydrolase | *Legionella adelaidensis* | 43 |
| Structure and packaging | tail | pVa21_110 | Putative tail assembly protein | *Cronobacter* phage CR5 | 39 |
| lysis |  | pVa21_119 | Putative lytic transglycosylase | *Pseudomonas* phage PaBG | 37 |
| Structure and packaging | tail | pVa21_120 | Putative tail fiber assembly protein | Aquamicrobium phage P14 | 62 |
| Structure and packaging | tail | pVa21_121 | Minor tail protein | *Erwinia* phage vB_EamM_Asesino | 34 |
| Structure and packaging | tail | pVa21_122 | Tail fiber assembly protein | *Yersinia* sp. FDAARGOS_228 | 35 |
| Structure and packaging | membrane | pVa21_123 | Putative membrane protein | *Erwinia* phage vB_EamM_Phobos | 26 |
| Structure and packaging | tail | pVa21_124 | Putative tail tip protein | *Erwinia* phage vB_EamM_Phobos | 26 |
| Structure and packaging | tail | pVa21_125 | Putative tail fiber protein | *Cronobacter* phage CR5 | 29 |
| Structure and packaging |  | pVa21_126 | Putative virion structural protein | *Cronobacter* phage CR5 | 30 |
| Structure and packaging |  | pVa21_127 | Putative virion structural protein | *Erwinia* phage vB_EamM_Caitlin | 44 |
| Nucleotide regulation | repair | pVa21_128 | Methyl-directed repair DNA adenine methylase | *Photobacterium* sp. J15 | 54 |
| Nucleotide regulation |  | pVa21_131 | Putative SMC domain-containing protein | *Erwinia* phage vB_EamM_Phobos | 32 |
| Nucleotide regulation | tRNA synthesis | pVa21_133 | Putative Appr-1-p processing protein | *Erwinia* phage vB_EamM_Huxley | 38 |
| lysis |  | pVa21_134 | Lytic transglycosylase | *Erwinia* phage PhiEaH1 | 37 |
| Structure and packaging |  | pVa21_136 | Putative virion structural protein | *Salmonella* phage SPN3US | 41 |
| Nucleotide regulation | breakdown of host DNA | pVa21_139 | Putative endodeoxyribonuclease | *Erwinia* phage vB_EamM_Caitlin | 37 |
| Nucleotide regulation | replication | pVa21_140 | Putative ribonuclease H | *Erwinia* phage vB_EamM_Phobos | 31 |
| Nucleotide regulation | repair | pVa21_148 | Putative UvsX protein | *Erwinia* phage vB_EamM_EarlPhillipIV | 39 |
| Structure and packaging |  | pVa21_151 | Putative virion structural protein | *Salmonella* phage SPN3US | 46 |
| Structure and packaging |  | pVa21_153 | Putative virion structural protein | *Erwinia* phage vB_EamM_EarlPhillipIV | 32 |
| Structure and packaging | packaging | pVa21_162 | Preprotein translocase subunit | *Acidobacteria* bacterium 13_1_20CM_3_53_8 | 56 |
| lysis |  | pVa21_165 | lytic transglycoslyase | *Erwinia* phage vB_EamM_Parshik | 26 |
| Nucleotide regulation |  | pVa21_166 | Putative DNA-directed RNA polymerase beta subunit | *Erwinia* phage vB_EamM_Huxley | 55 |
| Nucleotide regulation |  | pVa21_167 | Putative DNA-directed RNA polymerase beta subunit | *Cronobacter* phage CR5 | 43 |
| Structure and packaging |  | pVa21_171 | Putative virion structural protein | *Erwinia* phage vB_EamM_Asesino | 49 |
| Nucleotide regulation | repair | pVa21_173 | Putative DNA repair exonuclease | *Erwinia* phage vB_EamM_Caitlin | 41 |
| Structure and packaging |  | pVa21_180 | Putative virion structural protein | *Erwinia* phage vB_EamM_Phobos | 48 |
| Structure and packaging | tail | pVa21_181 | Putative tail sheath protein | *Salmonella* phage SPN3US | 48 |
| Structure and packaging |  | pVa21_183 | Putative virion structural protein | *Cronobacter* phage CR5 | 39 |
| Structure and packaging |  | pVa21_184 | Putative virion structural protein | *Erwinia* phage vB_EamM_Phobos | 50 |
| Structure and packaging |  | pVa21_185 | putative terminase large subunit | *Erwinia* phage vB_EamM_Caitlin | 45 |
| Structure and packaging |  | pVa21_198 | HNH endonuclease | *Vibrio harveyi* | 43 |
| Nucleotide regulation |  | pVa21_209 | Putative DNA-directed RNA polymerase beta subunit | *Pseudomonas* phage PhiPA3 | 35 |
| Nucleotide regulation | replication | pVa21_213 | Putative nuclease SbcCD D subunit | *Erwinia* phage vB_EamM_Phobos | 37 |
| Nucleotide regulation |  | pVa21_220 | Putative DNA-directed RNA polymerase beta subunit | *Erwinia* phage vB_EamM_Kwan | 29 |
| Nucleotide regulation |  | pVa21_221 | Putative DNA-directed RNA polymerase beta subunit | *Pseudomonas* phage 201phi2-1 | 29 |
| Nucleotide regulation | replication | pVa21_222 | Putative helicase | *Erwinia* phage vB_EamM_EarlPhillipI | 40 |
| Nucleotide regulation |  | pVa21_228 | Putative DNA-directed RNA polymerase beta subunit | *Salmonella* phage SPN3US | 44 |
| Nucleotide regulation |  | pVa21_231 | Putative B family DNA polymerase | *Erwinia* phage vB_EamM_EarlPhillipIV | 46 |
| Structure and packaging |  | pVa21_233 | Putative virion structural protein | *Erwinia* phage phiEaH2 | 33 |
| Structure and packaging |  | pVa21_236 | Putative virion structural protein | *Erwinia* phage vB_EamM_EarlPhillipIV | 40 |
| Structure and packaging |  | pVa21_237 | Putative virion structural protein | *Erwinia* phage vB_EamM_Huxley | 42 |
| Structure and packaging |  | pVa21_238 | Putative virion structural protein | *Erwinia* phage vB_EamM_EarlPhillipIV | 42 |
| Structure and packaging |  | pVa21_239 | Putative virion structural protein | *Erwinia* phage vB_EamM_Asesino | 34 |

| **Phage** | | **ACC. NO** | **Identity (%)** | | | **Gap (%)** | | |
| --- | --- | --- | --- | --- | --- | --- | --- | --- |
|  |  |  | **Terminase large** | **Major capsid** | **Whole geneome** | **Terminase large** | **Major capsid** | **Whole geneome** |
| **phiKZ-like phages** | **phiKZ** | AF399011 | 1130/2212 (51.1%) | 1192/2386 (50.0%) | 133142/284776 (46.80%) | 149/2212 (6.7%) | 260/2386 (10.9%) | 57220/284776  (20.10%) |
|  | **VP4B** | KC131130 | 1195/2411 (49.6%) | 1219/2422 (50.3%) | 120487/250105 (48.20%) | 367/2411 (15.2%) | 383/2422 (15.8%) | 32159/250105 (12.90%) |
|  | **pTD1** | AP017972 | 1121/2339 (47.9%) | 1199/2389 (50.2%) | 121533/252377 (48.20%) | 361/2339  (15.4%) | 314/2389 (13.1%) | 33480/252377 (13.30%) |
|  | **CR5** | JX094500 | 1183/2151 (55.0%) | 1269/2375 (53.4%) | 116892/244345 (47.80%) | 75/2151 (3.5%) | 226/2375 (9.5%) | 32703/244345 (13.40%) |
|  | **SPN3US** | JN641803 | 1263/2182 (57.9%) | 1256/2389 (52.6%) | 121141/252818 (47.90%) | 131/2182 (6.0%) | 209/2389 (8.7%) | 33225/252818 (13.10%) |
|  | **SEGD1** | KU726251 | 1266/2182 (58.0%) | 1183/2367 (50.0%) | 120949/252477 (47.90%) | 131/2182 (6.0%) | 399/2367 (16.9%) | 33495/252477 (13.30%) |
| **schizo T4-like phages** | **T4** | AF158101 | 1041/2177 (47.8%) | 999/2282 (43.8%) | 103491/233556 (44.30%) | 400/2177 (18.4%) | 730/2282 (32.0%) | 66211/233556 (28.30%) |
|  | **KVP40** | AY283928 | 1033/2158  (47.9%) | 1009/2277 (44.3%) | 123692/256088 (48.30%) | 392/2158 (18.2%) | 741/2277 (32.5%) | 35344/256088 (13.80%) |
|  | **phi-pp2** | JN849462 | 1034/2160 (47.9%) | 1009/2276 (44.3%) | 124400/257234 (48.40%) | 396/2160 (18.3%) | 739/2276 (32.5%) | 36049/257234 (14.00%) |
|  | **phi-ST2** | KT919973 | 1005/2152 (46.7%) | 1019/2280 (44.7%) | 125412/260057 (48.20%) | 380/2152 (17.7%) | 747/2280 (32.8%) | 37631/260057 (14.50%) |
|  | **phi-Grn1** | KT919972 | 1006/2152 (46.7%) | 1020/2278 (44.8%) | 124786/258608 (48.3%) | 380/2152 (17.7%) | 743/2278 (32.6%) | 36613/258608 (14.2%) |
|  | **VH7D** | KC131129 | 1007/2159 (46.6%) | 1020/2278 (44.8%) | 124042/257146 (48.20%) | 466/2159 (21.6%) | 743/2278 (32.6%) | 35330/257146 (13.70%) |
|  | **ValKK3** | KP671755 | 1032/2178 (47.4%) | 1022/2280 (44.8%) | 124392/258105 (48.20%) | 432/2178 (19.8%) | 747/2280 (32.8%) | 36124/258105 (14.00%) |

**Table S2** Comparative nucleotide analysis between the genomes of pVa-21 and phages analyzed in this study. The identity between the genomes was calculated using EMBOSS stretcher.

**Table S3** Core genes shared by the eight *Vibrio* phages analyzed in this study.

|  | **Phi KZ like phages** | |  | | **Schizo T4 like phages** | | | | | |  |
| --- | --- | --- | --- | --- | --- | --- | --- | --- | --- | --- | --- |
| **pVa-21** | **VP4B** | **pTD1** | **KVP40** | **phi-pp2** | | **phi-ST2** | **phi-Grn1** | **VH7D** | **ValKK3** | |  |
| hypothetical protein (AQT27943.1) | hypothetical protein (AGB07240.1) | phage protein (BAW98292.1) |  |  | |  |  |  |  | |  |
| putative virion structural protein (AQT27944.1) | hypothetical protein (AGB07174.1) | phage protein (BAW98291.1) |  |  | |  |  |  |  | |  |
| putative virion structural protein (AQT27946.1) | hypothetical protein (AGB07243.1) | phage protein (BAW98289.1) |  |  | |  |  |  |  | |  |
| putative helicase (AQT27951.1) | hypothetical protein (AGB07255.1) | DNA helicase, phage-associated (BAW98276.1) |  |  | |  |  |  |  | |  |
| putative major capsid protein (AQT27953.1) | hypothetical protein (AGB07257.1) | phage capsid and scaffold (BAW98274.1) |  |  | |  |  |  |  | |  |
| hypothetical protein (AQT27954.1) |  |  | hypothetical protein (NP_899585.1) |  | |  |  |  |  | |  |
| putative RNA polymerase beta subunit (AQT27955.1) | hypothetical protein (AGB07259.1) | phage protein (BAW98272.1) |  |  | |  |  |  |  | |  |
| putative virion structural protein (AQT27958.1) | hypothetical protein (AGB07267.1) | phage protein (BAW98264.1) |  |  | |  |  |  |  | |  |
| putativevirion structural protein (AQT27959.1) | hypothetical protein (AGB07268.1) | phage protein (BAW98263.1) |  |  | |  |  |  |  | |  |
| putative virion structural protein (AQT27961.1) | hypothetical protein (AGB07270.1) | phage protein (BAW98262.1) |  |  | |  |  |  |  | |  |
| putative crossover junction endodeoxyribonuclease (AQT27962.1) | hypothetical protein (AGB07271.1) | phage protein (BAW98261.1) |  |  | |  |  |  |  | |  |
| thymidylate synthase (AQT27999.1) | thymidylate synthase (AGB07147.1) | thymidylate synthase (BAW98385.1) | dMTP (thymidylate) synthase (NP_899279.1) | thymidylate synthase (AFN37265.1) | | thymidylate synthase (ALP47405.1) | thymidylate synthase (ALP47026.1) | thymidylate synthase (AGB07038.1) | thymidylate synthase (AJT61071.1) | |  |
| putative tail fiber protein (AQT28030.1) |  |  | long tail fiber, proximal subunit (NP_899629.1) | long tail fiber, proximal subunit (AFN37615.1) | | short tail fiber protein (ALP47338.1) | straight tail fiber (ALP46958.1) |  | long tail fiber proximal subunit (AJT61103.1) | |  |
| thymidylate kinase (AQT28041.1) |  | hypothetical protein (BAW98364.1) |  |  | |  |  |  |  | |  |
| deoxyuridine 5'-triphosphate nucleotidohydrolase (AQT28046.1) |  |  | deoxyuridine 5'-triphosphate nucleotidohydrolase (NP_889269.1) | deoxyuridine 5'-triphosphate nucleotidohydrolase (AFN37255.1) | | deoxyuridine 5'-triphosphate nucleotidohydrolase (ALP47489.1) | dUTP pyrophosphatase (ALP47106.1) | deoxyuridine 5'-triphosphate nucleotidohydrolase (AGB07028.1) | deoxyuridine 5'-triphosphate nucleotidohydrolase (AJT61082.1) | |  |
| minor tail protein (AQT28062.1) | hypothetical protein (AGB07224.1) | hypothetical protein (BAW98364.1) | prohead core protein (NP_899608.1) | prohead core protein (AFN37594.1) | |  |  |  |  | |  |
| putative membrane protein (AQT28064.1) |  |  |  |  | | tail fibers protein (ALP47361.1) | putative minor structural protein (ALP46984.1) | short tail fiber protein (AGB06989.1) | short tail fiber protein (AJT61137.1) | |  |
| putative tail tip protein (AQT28065.1) | hypothetical protein (AGB07276.1) | phage minor tail protein (BAW98255.1) |  |  | |  |  |  |  | |  |
| putative tail fiber protein (AQT28066.1) | hypothetical protein (AGB07278.1) | phage minor tail protein (BAW98254.1) |  |  | | tail fibers protein (ALP46337.1) | tail fibers protein (ALP46978.1) |  | long tail fier distal subunit (AJT61190.1) | |  |
| putative virion structural protein (AQT28067.1) | hypothetical protein (AGB07275.1) | phage protein (BAW98257.1) |  |  | | NADPH-dependent 7-cyano-7-deazaguanine reductase (ALP47397.1) |  | hypothetical protein (AGB06897.1) | 6-pyruvoyltetrahydropterin synthase-like protein (AJT60981.1) | |  |
| putative virion structural protein (AQT28068.1) | hypothetical protein (AGB07280.1) | phage protein (BAW98252.1) |  |  | |  |  |  |  | |  |
| putative SMC domain-containing protein (AQT28072.1) | hypothetical protein (AGB07281.1) | DNA double-strand break repair Rad50 ATPase (BAW 98251.1) | recombination endonuclease subunit (NP_899322.1) | recombination endonuclease subunit (AFN37305.1) | | hypothetical protein (ALP47439.1) | recombination-related endonuclease (ALP46964.1) | hypothetical protein (AGB06887.1) | hypothetical protein (AJT60885.1) | |  |
| hypothetical protein (AQT28076.1) | hypothetical protein (AGB07321.1) | phage protein (BAW98418.1) |  |  | |  |  |  |  | |  |
| putative virion structural protein (AQT28077.1) | hypothetical protein (AGB07322.1) | phage protein (BAW98417.1) |  |  | |  |  |  |  | |  |
| putative endodeoxyribonuclease (AQT28080.1) |  |  | exonuclease (NP_899271.1) | exonuclease A (AFN37257.1) | | exonuclease A (ALP47434.1) | exonuclease A (ALP47053.1) | exodeoxyribonuclease (AGB07030.1) | hypothetical protein (AJT6100.1) | |  |
| putative ribonuclease H (AQT28081.1) | hypothetical protein (AGB07115.1) | ribonuclease HI (BAW98241.1) |  |  | |  |  |  |  | |  |
| putative UvsX protein (AQT28089.1) | hypothetical protein (AGB07117.1) | phage protein (BAW98413.1) |  |  | |  |  |  |  | |  |
| putative virion structural protein (AQT28092.1) | hypothetical protein (ABG07119.1) | phage protein (BAW98411.1) |  |  | |  |  |  |  | |  |
| putative virion structural protein (AQT28094.1) | hypothetical protein (AGB07121.1) | phage protein (BAW98409.1) |  |  | |  |  |  |  | |  |
| hypothetical protein (AQT28097.1) |  |  |  |  | | RNA polymerase-ADP-ribosyltransferase Alt (ALP47357.1) | RNA polymerase-ADP-ribosyltransferase Alt (ALP46977.1) |  | RNA polymerase-ADP-ribosyltransferase (AJT61043.1) | |  |
| hypothetical protein (AQT28105.1) | hypothetical protein (AGB07127.1) | phage protein (BAW98404.1) |  |  | |  |  |  |  | |  |
| lytic transglycosylase (AQT28106.1) | hypothetical protein (AGB07128.1) | phage endolysin (BAW98403.1) |  |  | | inhibitor of prohead protease (ALP47488.1) | inhibitor of prohead protease (ALP47105.1) | hypothetical protein (AGB07005.1) | prohead protease inhibitor (AJT61120.1) | |  |
| putative DNA-directed RNA polymerase beta subunit (AQT28107.1) | hypothetical protein (AGB07129.1) | phage protein (BAW98402.1) |  |  | |  |  |  |  | |  |
| putative DNA-directed RNA polymerase beta subunit (AQT28108.1) | hypothetical protein (AGB07131.1) | phage protein (BAW98401.1) |  |  | |  |  |  |  | |  |
| hypothetical protein (AQT28111.1) | hypothetical protein (AGB07135.1) | phage protein (BAW98397.1) |  |  | |  |  |  |  | |  |
| putative virion structural protein (AQT28112.1) | hypothetical protein (AGB07136.1) | phage protein (BAW98396.1) |  |  | |  |  |  |  | |  |
| putative virion structural protein (AQT28121.1) | hypothetical protein (AGB07158.1) | phage protein (BAW98374.1) |  |  | |  |  |  |  | |  |
| putative tail sheath protein (AQT28122.1) | hypothetical protein (AGB07159.1) | phage protein (BAW98373.1) |  |  | |  |  |  |  | |  |
| hypothetical protein (AQT28123.1) | hypothetical protein (AGB07160.1) | phage protein (BAW98372.1) |  |  | |  |  |  |  | |  |
| putative virion structural protein (AQT28124.1) | hypothetical protein (AGB07161.1) | phage protein (BAW98371.1) |  |  | |  |  |  |  | |  |
| putative virion structural protein (AQT28125.1) | hypothetical protein (AGB07162.1) | phage protein (BAW98370.1) |  |  | |  |  |  |  | |  |
| putative terminase large subunit (AQT28126.1) | hypothetical protein (AGB07167.1) | phage protein (BAW98365.1) | large terminase protein (NP_899601.1) | phage terminase large subunit (AFN37587.1) | | terminase large subunit (ALP46351.1) | terminase large subunit (ALP46971.1) | large terminase protein (AGB06996.1) | large terminase protein (AJT61130.1) | |  |
| hypothetical protein (AQT28131.1) | hypothetical protein (AGB07168.1) | phage protein (BAW98363.1) |  |  | |  |  |  |  | |  |
| hypothetical protein (AQT28140.1) | hypothetical protein (AGB07169.1) | phage protein (BAW98362.1) |  |  | |  |  |  |  | |  |
| hypothetical protein (AQT28146.1) | hypothetical protein (AGB07187.1) | phage protein (BAW98343.1) |  |  | |  |  |  |  | |  |
| hypothetical protein (AQT28147.1) | hypothetical protein (AGB07189.1) | phage protein (BAW98341.1) |  |  | |  |  |  |  | |  |
| hypothetical protein (AQT28149.1) | hypothetical protein (AGB07191.1) | phage protein (BAW98339.1) |  |  | |  |  |  |  | |  |
| putative DNA-directecd RNA polymerase beta subunit (AQT28150.1) | hypothetical protein (AGB07192.1) | phage protein (BAW98338.1) |  |  | |  |  |  |  | |  |
| hypothetical protein (AQT28151.1) | hypothetical protein (AGB07194.1) | phage protein (BAW98337.1) |  |  | |  |  |  |  | |  |
| putative nuclease SbcCD D subunit (AQT28154.1) | hypothetical protein (AGB07199.1) | phage protein (BAW98332.1) |  |  | |  |  |  |  | |  |
| hypothetical protein (AQT28155.1) | hypothetical protein (AGB07200.1) | phage protein (BAW98331.1) |  |  | |  |  |  |  | |  |
| hypothetical protein (AQT28156.1) | hypothetical protein (AGB07201.1) | phage protein (BAW98330.1) |  |  | |  |  |  |  | |  |
| hypothetical protein (AQT28157.1) | hypothetical protein (AGB07202.1) | phage protein (BAW98329.1) |  |  | |  |  |  |  | |  |
| hypothetical protein (AQT28158.1) | hypothetical protein (AGB07203.1) | phage protein (BAW98328.1) | moaA/nifB/pqqE family protein (NP_899507.1) |  | |  |  |  |  | |  |
| hypothetical protein (AQT28159.1) | hypothetical protein (AGB07204.1) | phage protein (BAW98327.1) |  |  | |  |  |  |  | |  |
| hypothetical protein (AQT28160.1) | hypothetical protein (AGB07206.1) | hypothetical protein (BAW98325.1) |  |  | |  |  |  |  | |  |
| putative DNA-directed RNA polymerase beta subunit (AQT28161.1) | hypothetical protein (AGB07207.1) | phage protein (BAW98324.1) |  |  | |  |  |  |  | |  |
| putative DNA-directed RNA polymerase beta subunit (AQT28162.1) | bifunctional DNA-directed RNA polymerase subunit beta'-beta'' (AGB07208.1) | phage terminase, large subunit @ intein-containing (BAW98323.1) |  |  | |  |  |  |  | |  |
| putative helicase (AQT28163.1) | hypothetical protein (AGB07209.1) | phage terminase, large subunit @ intein-containing (BAW98322.1) |  |  | |  |  |  |  | |  |
| hypothetical protein (AQT28166.1) | hypothetical protein (AGB07217.1) | phage protein (BAW98315.1) |  |  | |  |  |  |  | |  |
| putative DNA-directed RNA polymerase beta subunit (AQT28169.1) | hypothetical protein (AGB07214.1) | phage protein (BAW98318.1) |  |  | |  |  |  |  | |  |
| putative B family DNA polymerase (AQT28172.1) | hypothetical protein (AGB07221.1) | phage protein (BAW98311.1) |  |  | |  |  |  |  | |  |
| putative virion structural protein (AQT28174.1) | hypothetical protein (AGB07166.1) | phage protein (BAW98310.1) |  |  | |  |  |  |  | |  |
| putative virion structural protein (AQT28177.1) | hypothetical protein (AGB07226.1) | phage protein (BAW98306.1) |  |  | |  |  |  |  |  |  |
| putative virion structural protein (AQT28178.1) | hypothetical protein (AGB07227.1) | phage protein (BAW98305.1) |  |  | |  |  |  |  |  |  |
| putative virion structural protein (AQT28179.1) | hypothetical protein (AGB07228.1) | phage capsid and scaffold (BAW98304.1) |  |  | |  | hypothetical protein (ALP47149.1) | hypothetical protein (AGB06886.1) |  |  |  |
| hypothetical protein (AQT28182.1) | hypothetical protein (AGB07233.1) | hypothetical protein (BAW98293.1) |  |  | |  |  |  |  |  |  |
| dihydrofolate reductase (AQT28183.1) | dihydrofolate reductase (AGB07145.1) | dihydrofolate reductase (BAW98387.1) | dihydrofolate reductase (NP_889254.1) | dihydrofolate reductase (AFN37240.1) | | dihydrofolate reductase (ALP47469.1) | dihydrofolate reductase (ALP47088.1) | dihydrofolate reductase (AGB07013.1) | dihydrofolate reductase (AJT61097.1) | | |
